# *N*′-(Propan-2-yl­idene)nicotinohydrazide

**DOI:** 10.1107/S1600536809034734

**Published:** 2009-09-05

**Authors:** Feng-Yu Bao, Yu-Xia Zhang, Ying-Xia Zhou, Hai-Yan Zhang

**Affiliations:** a Department of Applied Chemistry, College of Sciences, Henan Agricultural University, Zhengzhou 450002, People’s Republic of China; b Sanonda Zhengzhou Pesticide Co Ltd, Zhengzhou 450009, People’s Republic of China

## Abstract

Crystals of the title compound, C_9_H_11_N_3_O, were obtained from a condensation reaction of nicotinohydrazide and acetone. In the mol­ecular structure, the pyridine ring is oriented at a dihedral angle of 36.28 (10)° with respect to the amide plane. In the crystal structure, mol­ecules are linked *via* N—H⋯O hydrogen bonds, forming chains.

## Related literature

For applications of Schiff base compounds, see: Kahwa *et al.* (1986 ▶); Santos *et al.* (2001 ▶).
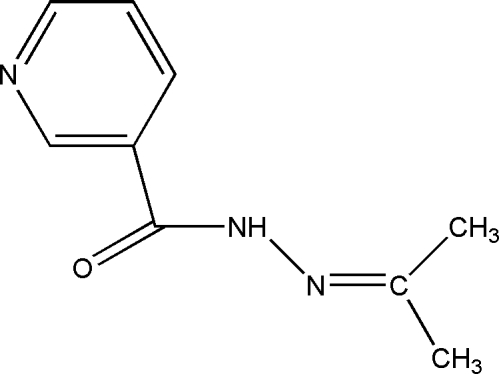


## Experimental

### 

#### Crystal data

C_9_H_11_N_3_O
*M* *_r_* = 177.21
Monoclinic, 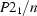

*a* = 7.5439 (4) Å
*b* = 18.0292 (9) Å
*c* = 7.6172 (4) Å
β = 115.937 (3)°
*V* = 931.67 (8) Å^3^
*Z* = 4
Mo *K*α radiation
μ = 0.09 mm^−1^
*T* = 296 K
0.42 × 0.21 × 0.12 mm

#### Data collection

Bruker SMART CCD area-detector diffractometer
Absorption correction: multi-scan (*SADABS*; Bruker, 1998 ▶) *T*_min_ = 0.978, *T*_max_ = 0.990
14297 measured reflections
2172 independent reflections
1301 reflections with *I* > 2σ(*I*)
*R*_int_ = 0.046

#### Refinement

*R*[*F* ^2^ > 2σ(*F* ^2^)] = 0.053
*wR*(*F* ^2^) = 0.161
*S* = 1.02
2172 reflections
120 parameters
H-atom parameters constrained
Δρ_max_ = 0.21 e Å^−3^
Δρ_min_ = −0.22 e Å^−3^

### 

Data collection: *SMART* (Bruker, 1998 ▶); cell refinement: *SAINT* (Bruker, 1998 ▶); data reduction: *SAINT*; program(s) used to solve structure: *SHELXTL* (Sheldrick, 2008 ▶); program(s) used to refine structure: *SHELXTL*; molecular graphics: *SHELXTL*; software used to prepare material for publication: *SHELXTL*.

## Supplementary Material

Crystal structure: contains datablocks global, I. DOI: 10.1107/S1600536809034734/xu2604sup1.cif

Structure factors: contains datablocks I. DOI: 10.1107/S1600536809034734/xu2604Isup2.hkl

Additional supplementary materials:  crystallographic information; 3D view; checkCIF report

## Figures and Tables

**Table 1:** Hydrogen-bond geometry (Å, °)

*D*—H⋯*A*	*D*—H	H⋯*A*	*D*⋯*A*	*D*—H⋯*A*
N2—H2*A*⋯O^i^	0.86	2.08	2.9136 (18)	162

Symmetry code: (i) 
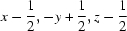
.

**Fig. 1.**
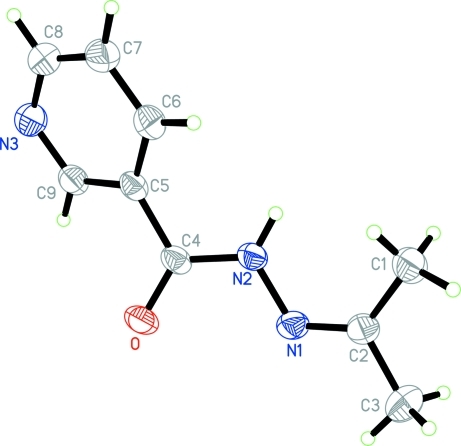
The molecular structure of the title compound. Displacement ellipsoids are drawn at the 30% probability level.

## supplementary crystallographic information

### Comment

The chemistry of Schiff bases has attracted a great deal of interest in recent years. These compounds play an important role in the development of various proteins and enzymes (Kahwa *et al.*, 1986; Santos *et al.*, 2001). As part of our interest in the coordination chemistry of Schiff bases, we have synthesized the title compound and report here its crystal structure.

IN the molecular structure (Fig. 1), the pyridine ring is oriented with respect to N2/C4/O plane with a dihedral angle of 36.28 (10)°. In the crystal structure intermolecular N—H···O hydrogen bonding links the molecules to form the one-dimensional chains (Table 1).

### Experimental

Nicotinohydrazide (1 mmol, 0.137 g) was dissolved in anhydrous ethanol (15 ml). The mixture was stirred for several min at 351 K, then the acetone (1 mmol, 0.058 g) in ethanol (8 ml) was added dropwise and the mixture was stirred at refluxing temperature for 2 h. The solid product was isolated and recrystallized from methanol. Colourless single crystals were obtained after 3 d.

### Refinement

All H atoms were positioned geometrically and refined as riding with C—H = 0.93 (aromatic), 0.96 Å (methyl) and N—H = 0.86 Å. *U*_iso_(H) = 1.5*U*_eq_(C) for methyl H atoms and *U*_iso_(H) = 1.2*U*_eq_(C,*N*) for the others.

### Crystal data

**Table:**

C_9_H_11_N_3_O	*F*(000) = 376
*M**_r_* = 177.21	*D*_x_ = 1.263 Mg m^−^^3^
Monoclinic, *P*2_1_/*n*	Mo *K*α radiation, λ = 0.71073 Å
Hall symbol: -P 2yn	Cell parameters from 3071 reflections
*a* = 7.5439 (4) Å	θ = 2.3–27.0°
*b* = 18.0292 (9) Å	µ = 0.09 mm^−^^1^
*c* = 7.6172 (4) Å	*T* = 296 K
β = 115.937 (3)°	Block, colourless
*V* = 931.67 (8) Å^3^	0.42 × 0.21 × 0.12 mm
*Z* = 4	

### Data collection

**Table:**

Bruker SMART CCD area-detector diffractometer	2172 independent reflections
Radiation source: fine-focus sealed tube	1301 reflections with *I* > 2σ(*I*)
graphite	*R*_int_ = 0.046
ω scans	θ_max_ = 27.7°, θ_min_ = 2.3°
Absorption correction: multi-scan (*SADABS*; Bruker, 1998)	*h* = −9→9
*T*_min_ = 0.978, *T*_max_ = 0.990	*k* = −23→23
14297 measured reflections	*l* = −9→9

### Refinement

**Table:**

Refinement on *F*^2^	Primary atom site location: structure-invariant direct methods
Least-squares matrix: full	Secondary atom site location: difference Fourier map
*R*[*F*^2^ > 2σ(*F*^2^)] = 0.053	Hydrogen site location: inferred from neighbouring sites
*wR*(*F*^2^) = 0.161	H-atom parameters constrained
*S* = 1.02	*w* = 1/[σ^2^(*F*_o_^2^) + (0.0882*P*)^2^ + 0.0683*P*] where *P* = (*F*_o_^2^ + 2*F*_c_^2^)/3
2172 reflections	(Δ/σ)_max_ < 0.001
120 parameters	Δρ_max_ = 0.21 e Å^−^^3^
0 restraints	Δρ_min_ = −0.22 e Å^−^^3^

### Special details

**Table:**

Geometry. All e.s.d.'s (except the e.s.d. in the dihedral angle between two l.s. planes) are estimated using the full covariance matrix. The cell e.s.d.'s are taken into account individually in the estimation of e.s.d.'s in distances, angles and torsion angles; correlations between e.s.d.'s in cell parameters are only used when they are defined by crystal symmetry. An approximate (isotropic) treatment of cell e.s.d.'s is used for estimating e.s.d.'s involving l.s. planes.
Refinement. Refinement of *F*^2^ against ALL reflections. The weighted *R*-factor *wR* and goodness of fit *S* are based on *F*^2^, conventional *R*-factors *R* are based on *F*, with *F* set to zero for negative *F*^2^. The threshold expression of *F*^2^ > σ(*F*^2^) is used only for calculating *R*-factors(gt) *etc*. and is not relevant to the choice of reflections for refinement. *R*-factors based on *F*^2^ are statistically about twice as large as those based on *F*, and *R*- factors based on ALL data will be even larger.

### Fractional atomic coordinates and isotropic or equivalent isotropic displacement parameters (Å^2^)

**Table:**

	*x*	*y*	*z*	*U*_iso_*/*U*_eq_	
N1	0.6021 (2)	0.20018 (9)	0.3243 (2)	0.0526 (4)	
N2	0.5607 (2)	0.23097 (8)	0.1426 (2)	0.0498 (4)	
H2A	0.4896	0.2078	0.0361	0.060*	
N3	0.5259 (3)	0.44511 (10)	−0.2362 (3)	0.0683 (5)	
O	0.74766 (19)	0.33093 (7)	0.29001 (19)	0.0640 (4)	
C1	0.5162 (3)	0.07664 (11)	0.1687 (3)	0.0707 (6)	
H1A	0.3751	0.0725	0.1065	0.106*	
H1B	0.5731	0.0290	0.2184	0.106*	
H1C	0.5612	0.0935	0.0755	0.106*	
C2	0.5773 (2)	0.13080 (11)	0.3328 (3)	0.0522 (5)	
C3	0.6197 (3)	0.10093 (13)	0.5301 (3)	0.0748 (6)	
H3A	0.6712	0.1398	0.6252	0.112*	
H3B	0.7147	0.0617	0.5627	0.112*	
H3C	0.5003	0.0821	0.5293	0.112*	
C4	0.6361 (2)	0.29792 (10)	0.1407 (3)	0.0470 (5)	
C5	0.5842 (2)	0.33173 (9)	−0.0540 (2)	0.0448 (4)	
C6	0.5646 (3)	0.29200 (11)	−0.2158 (3)	0.0553 (5)	
H7A	0.5770	0.2406	−0.2101	0.066*	
C7	0.5262 (3)	0.32960 (13)	−0.3867 (3)	0.0649 (6)	
H8A	0.5129	0.3040	−0.4978	0.078*	
C8	0.5083 (3)	0.40459 (13)	−0.3895 (3)	0.0678 (6)	
H9A	0.4821	0.4292	−0.5055	0.081*	
C9	0.5659 (3)	0.40808 (11)	−0.0721 (3)	0.0547 (5)	
H10A	0.5827	0.4353	0.0377	0.066*	

### Atomic displacement parameters (Å^2^)

**Table:**

	*U*^11^	*U*^22^	*U*^33^	*U*^12^	*U*^13^	*U*^23^
N1	0.0529 (9)	0.0484 (9)	0.0449 (9)	0.0063 (7)	0.0108 (7)	0.0040 (7)
N2	0.0472 (8)	0.0448 (9)	0.0428 (8)	−0.0020 (7)	0.0062 (6)	−0.0001 (7)
N3	0.0776 (12)	0.0534 (11)	0.0617 (11)	−0.0046 (8)	0.0191 (9)	0.0067 (9)
O	0.0620 (8)	0.0498 (8)	0.0513 (8)	−0.0055 (6)	−0.0019 (6)	−0.0041 (6)
C1	0.0713 (13)	0.0476 (12)	0.0699 (14)	0.0045 (10)	0.0092 (11)	0.0022 (10)
C2	0.0411 (9)	0.0499 (11)	0.0542 (11)	0.0086 (8)	0.0103 (8)	0.0066 (9)
C3	0.0817 (14)	0.0678 (14)	0.0718 (15)	0.0150 (12)	0.0306 (12)	0.0183 (12)
C4	0.0382 (8)	0.0406 (10)	0.0480 (10)	0.0027 (7)	0.0056 (7)	−0.0036 (8)
C5	0.0341 (8)	0.0432 (10)	0.0481 (11)	−0.0022 (7)	0.0096 (7)	−0.0036 (8)
C6	0.0563 (11)	0.0473 (11)	0.0605 (13)	−0.0027 (8)	0.0239 (9)	−0.0063 (9)
C7	0.0699 (13)	0.0732 (15)	0.0559 (13)	−0.0130 (11)	0.0315 (10)	−0.0100 (11)
C8	0.0732 (13)	0.0705 (15)	0.0555 (13)	−0.0120 (11)	0.0244 (11)	0.0041 (11)
C9	0.0528 (10)	0.0466 (11)	0.0532 (11)	−0.0041 (8)	0.0127 (8)	−0.0030 (9)

### Geometric parameters (Å, °)

**Table:**

N1—C2	1.271 (2)	C3—H3A	0.9600
N1—N2	1.394 (2)	C3—H3B	0.9600
N2—C4	1.337 (2)	C3—H3C	0.9600
N2—H2A	0.8600	C4—C5	1.489 (2)
N3—C9	1.330 (2)	C5—C6	1.376 (2)
N3—C8	1.334 (3)	C5—C9	1.384 (2)
O—C4	1.232 (2)	C6—C7	1.382 (3)
C1—C2	1.492 (3)	C6—H7A	0.9300
C1—H1A	0.9600	C7—C8	1.358 (3)
C1—H1B	0.9600	C7—H8A	0.9300
C1—H1C	0.9600	C8—H9A	0.9300
C2—C3	1.493 (3)	C9—H10A	0.9300
			
C2—N1—N2	117.96 (15)	H3B—C3—H3C	109.5
C4—N2—N1	117.32 (14)	O—C4—N2	123.21 (17)
C4—N2—H2A	121.3	O—C4—C5	119.84 (16)
N1—N2—H2A	121.3	N2—C4—C5	116.93 (14)
C9—N3—C8	116.26 (18)	C6—C5—C9	117.46 (17)
C2—C1—H1A	109.5	C6—C5—C4	123.87 (16)
C2—C1—H1B	109.5	C9—C5—C4	118.56 (16)
H1A—C1—H1B	109.5	C5—C6—C7	118.99 (18)
C2—C1—H1C	109.5	C5—C6—H7A	120.5
H1A—C1—H1C	109.5	C7—C6—H7A	120.5
H1B—C1—H1C	109.5	C8—C7—C6	118.78 (19)
N1—C2—C1	126.87 (17)	C8—C7—H8A	120.6
N1—C2—C3	115.77 (18)	C6—C7—H8A	120.6
C1—C2—C3	117.34 (18)	N3—C8—C7	124.1 (2)
C2—C3—H3A	109.5	N3—C8—H9A	117.9
C2—C3—H3B	109.5	C7—C8—H9A	117.9
H3A—C3—H3B	109.5	N3—C9—C5	124.38 (18)
C2—C3—H3C	109.5	N3—C9—H10A	117.8
H3A—C3—H3C	109.5	C5—C9—H10A	117.8

### Hydrogen-bond geometry (Å, °)

**Table:**

*D*—H···*A*	*D*—H	H···*A*	*D*···*A*	*D*—H···*A*
N2—H2A···O^i^	0.86	2.08	2.9136 (18)	162

Symmetry codes: (i) *x*−1/2, −*y*+1/2, *z*−1/2.
